# Is Ignorance of Climate Change Culpable?

**DOI:** 10.1007/s11948-016-9835-5

**Published:** 2016-11-28

**Authors:** Philip Robichaud

**Affiliations:** Philosophy Department, Vrije Universiteit Amsterdam, de Boelelaan 1105, 1081 HV Amsterdam, The Netherlands

**Keywords:** Climate change, Culpable ignorance, Moral responsibility, Blame

## Abstract

Sometimes ignorance is an excuse. If an agent did not know and could not have known that her action would realize some bad outcome, then it is plausible to maintain that she is not to blame for realizing that outcome, even when the act that leads to this outcome is wrong. This general thought can be brought to bear in the context of climate change insofar as we think (a) that the actions of individual agents play some role in realizing climate harms and (b) that these actions are apt targets for being considered right or wrong. Are agents who are ignorant about climate change and the way their actions contribute to it excused because of their ignorance, or is their ignorance culpable? In this paper I examine these questions from the perspective of recent developments in the theories of responsibility for ignorant action and characterize their verdicts. After developing some objections to existing attempts to explore these questions, I characterize two influential theories of moral responsibility and discuss their implications for three different types of ignorance about climate change. I conclude with some recommendations for how we should react to the face of the theories’ conflicting verdicts. The answer to the question posed in the title, then, is: “Well, it’s complicated.”

## The Problem

Sometimes ignorance is an excuse. If an agent did not know and could not have known that her action would bring about some bad outcome, it is plausible to maintain that she is not to blame for realizing that outcome. Relatedly, if an agent did not know and could not have known that her action was morally impermissible (perhaps in virtue of the fact that it realized some bad outcome), it is plausible to maintain that she is not to blame for acting wrongly. These general claims can be brought to bear in the context of climate change insofar as we think (a) that the actions of individual agents play some role in realizing climate harms or (b) that these actions are apt targets for being considered right or wrong. For example, an energy company CEO might be ignorant of the fact that her choice to implement some strategy will result in the avoidable production of billions of tons of carbon dioxide. Similarly, someone who is skeptical of that impact of human activities on climate might opt for a polluting energy provider instead of a similarly priced carbon–neutral provider. It is plausible that both of these agents played some role in the manifestation of future climate harms and it is even more plausible they acted impermissibly. Establishing each of these claims definitively would be an interesting and significant task, as would the defense of a moral theory that entails both that the actions of individuals bear moral significance in the context of climate ethics and, perhaps relatedly, that there are individual moral obligations to perform certain climate-harm-mitigating actions. As I discuss below, others have mounted detailed and complex arguments for these empirical and normative claims, and I for my purposes I will just stipulate that they hold. My focus here is the distinct question of whether agents who act on the basis of ignorance of these claims are excused by their ignorance. For example, does the CEO’s ignorance about the impact of her decision excuse her from blame? Does the climate science skeptic’s ignorance about climate science get her off the hook?

In this paper I examine these questions from the perspective of recent developments in the theories of responsibility for ignorant action and characterize their verdicts. My first task is to discuss three assumptions that I make in the paper and introduce a number of important clarifications. The first assumption concerns the worry that individuals are causally impotent in an important respect. I will assume that the actions of individuals are capable of realizing climate change-related harms through their actions. Because the harms related to the extreme weather events that are linked to human-induced climate change are only diffusely connected with individual actions, it is not obvious that individuals are capable of harming the climate at all, much less wrongfully bringing about avoidable climate harms. Given this, it is not clear that we are justified in treating individual actions as the relevant unit of moral evaluation, and so it is not clear that the question of whether individuals are blameworthy for wrongfully, though ignorantly, harming the environment will even arise. One way of addressing this worry is mathematical. David Frame calculated that over the course of her life, someone from a wealthy county will contribute over 800 tons of carbon dioxide to the atmosphere which will result in about half a billionth of a degree of warming (forthcoming). He calculates that his amount of warming will shorten people’s lives by several months and that each year of such emissions will result in shortening lives by one or two days. Another solution to the causal impotence problem has been developed by Shelly Kagan who argues that individual actions can make a difference, just so long as the agent’s action is a member of a set of actions which taken together suffice to bring about some outcome, which in this case will be some magnification of climate-change related harms (2011).^1^ For the purposes of this paper, I will assume that some such solution is successful and that the harm brought about by (sets of) individual actions is indeed significant and can function as a basis on which to ground the claim that these (sets of) actions are morally impermissible.^2^ Of course, further embedded empirical assumptions here are that anthropogenic climate change is indeed occurring and that the mechanisms by which it is occurring are such that its course can be affected by future human action. Like most of my interlocutors in the climate ethics literature, some such empirical background is taken as starting point for ethical inquiry, and I do the same.

The second assumption is a straightforwardly normative one—I assume that certain individuals have moral obligations to perform particular actions (or omissions) on grounds that these actions are in some way relevant to the eventual realization of climate harms. This is without question a substantive assumption, since even if one grants that individuals contribute *causally* to the realization of climate harms, this does not alone settle the normative question about whether we have individual obligations not to. Indeed, Sinnott-Armstrong has argued that there is no plausible moral principle from which it follows that individuals (as opposed to, say, nations) have moral obligations to diminish their carbon footprints (2010) and Johnson has argued that climate change has the structure of a tragedy of the commons and that in that context individuals lack moral obligations to curb their emissions (2003).^3^ Others have argued that individuals do in fact have such obligations. Broome defends the claim that individuals have “duties of justice” to minimize our carbon footprint and to engage in political actions that might result in policy changes that mitigate emissions (2012). Cripps argues that we have individual moral obligations to form collectives that are capable of addressing climate change on a larger scale (2013).^4^ Finally Jamieson (2007) and Sandberg (2011) have each argued that individuals should inculcate “green virtues”, which would involve character traits that dispose one to minimize emissions. Along the lines suggested by Hursthouse (1998), one could straightforwardly derive claims about the wrongness of certain individual actions from these virtues. It is obviously beyond my scope to adjudicate this debate, and for the purposes of exploring the question of whether individuals are blameworthy for their easily avoidable emissions, their failure to form collectives that would be able to realise large-scale emissions reductions, or their failure to inculcate green virtues, I will assume that the relevant individual moral obligations have some normative foundation.

Thirdly and finally, I assume that many actions that result in climate harms are performed from a state of ignorance about certain relevant empirical and normative claims. The claims about which the agents in question may be ignorant includes at one or another of the following:

- Ignorance that climate change is occurring
- Ignorance that one’s actions contribute in some way to climate change
- Ignorance that climate change is bad
- Ignorance that one has any individual obligations in the face of the badness of climate change
- Ignorance about how to meet such obligations
- Ignorance about the moral significance of mitigating climate change.
- Ignorance about which moral theory makes it true that climate change has moral significance

Agents who violate an obligation to choose a low-emissions option but who are ignorant in any of these ways may act differently if their ignorance was corrected. For example, if Ariel believes that climate change is not occurring he might disregard every opportunity to mitigate his carbon footprint. If his ignorance was corrected, he might conduct his affairs completely differently, even if none of his other beliefs or desires also changed. We can imagine that he had all manner of conditional intentions to use low-carbon energy sources if he was convinced that climate change was actually occurring, but that these intentions were blocked by his belief that it is not. Similarly, imagine Bella believes that climate change is occurring but falsely believes that it is actually a phenomenon to be welcomed. Again, this belief may be what hinders her from making choices that are climate change mitigating.^5^ Finally, imagine Chester believes both that climate change is occurring and that it is bad, but believes falsely that he has no individual moral obligations with respect to mitigating it. Perhaps, this is because he thinks the obligations to do something about it fall entirely on collective agents like national governments or international coalitions. If he were to see that indeed he does have individual obligations, Chester might make many different decisions. Thus, Ariel, Bella, and Chester all act ignorantly in ways that exacerbate climate change, which raises the question of whether they are culpable either for their ignorance or their actions based on this ignorance.

Importantly, these different species of ignorance can be roughly classified into non-moral ignorance [(a), (b), and (e)], which is ignorance about certain morally relevant empirical claims, and moral ignorance [(c), (d), (f), and (g)], which is ignorance about certain moral claims. Chester’s ignorance is probably of the latter category. His false belief that only governments are obligated to tackle these issues might be based on an inaccurate picture of how demanding individual morality is. In what follows, my focus will be cases of non-moral ignorance because they avoid certain complications associated with the way quality-of-will theories of moral responsibility, which is one class of theories that I discuss below, treat cases of moral ignorance. A more complete treatment of these issues would release this restriction and examine cases such as Chester’s.

Notice, however, that even non-moral ignorance such as Ariel’s and Bella’s may lead them to have false moral beliefs as well. Their ignorance of non-moral claims may lead them to think certain actions are morally permissible when they aren’t. For example, Ariel may infer from her belief that climate change is not occurring to the false belief that she has no moral obligation to take steps to mitigate it. Why, after all, would morality require one to mitigate something that is not occurring? Call this kind of moral ignorance *derivative moral ignorance*. It is ignorance about the truth of a moral claim that derives from non-moral ignorance. This is to be contrasted with Chester’s false belief that he has no moral obligation to take steps to mitigate climate change. Since his ignorance of truth of the moral claim that he does have obligations to take such steps does not derive from non-moral ignorance at all, call this *non*-*derivative moral ignorance*. Thus, even though I am restricting my attention to non-moral ignorance, I am also concerned by implication with cases of derivative moral ignorance as well.

With these three assumptions on the table, my target question should be clearer: When someone is ignorant about some relevant non-moral issue and on the basis of this ignorance violates an obligation to mitigate harms associated with climate change, is she blameworthy for her wrong action or its bad outcomes? Before discussing several different cases of ignorance, I must make two important clarifications. The first is that the sense of moral responsibility at issue is sometimes called backward-looking moral responsibility given that it is concerned with the question of whether certain agents deserve praise or blame for their actions. I am not addressing the question of whether individuals have forward-looking, substantive responsibilities or obligations to do their part to mitigate climate change. As stated above, I am simply stipulating that individuals have such responsibilities and obligations. My target, which is shared by all of the authors whose work I discuss below, is whether we are justified in making a further claim about such individuals, namely that they are backward-looking-responsible for their failures to make efforts to mitigate harmful anthropogenic changes in the climate.^6^ Second, I will focus only on the issue of whether agents are morally responsible for their ignorant actions or omissions. I set aside the question of whether agents are blameworthy, in an epistemic sense, simply for being ignorant in any of the ways described above.

One might think that given these assumptions and clarifications that my focus is overly narrow. I do not deny that I have indeed taken some interesting and important questions off the table, and I believe that a complete treatment of these issues is desirable. But, I take it that there are several independent but related reasons to proceed as I do. The first is simply that I think the arguments below offer an improvement upon the existing literature on this issue, all of which begins from a similar set of assumptions. If I were to relax these assumptions or adopt a substantially different set of assumptions, this project could not be seen as an advance on these discussions. Second, in addition to thinking that it is bad or wrong for individuals to contribute to climate harms, we also think that certain individuals can be blameworthy for wrongfully failing to do something that seeks to mitigate climate harms. Our moral discourse with regard to this and other areas is richer than merely judgments and discussions concerning what people morally should and should not do. Once we accept that individual obligations exist it is only natural to inquire into whether agents meet the conditions of moral responsibility with respect to their actions. Once this further question is posed and once it is noticed that the agent’s knowledge about what she has done (or lack thereof) is relevant to her responsibility for it, we must engage in the sort of inquiry that follows. Third, and most importantly, the question of responsibility that I take up is a vital one for any theory that indexes the strength of forward-looking responsibilities to, say, facilitate adaption to a warmer climate or to compensate those who suffer from climate harms to claims about whether individuals are to blame for wrongfully contributing to the problem. For example, Baer argues that fairness in distribution of adaptation costs turns on *inter alia* the issue of moral responsibility for climate harms (2006). Indeed, he speaks directly to the main topic of this paper when he says: “Although I argued that ignorance of harmful effects does not eliminate ethical and legal liability, there is little doubt that it is a relevant factor in considering exactly how liability should be limited” (p. 139). So, although I hew to what seems like a narrow set of issues in this paper, they should be of broad interest both because they advance a burgeoning topic in the climate change literature and because of their relevance to anyone who thinks that we have individual moral obligations to contribute to the mitigation of climate change harms and, perhaps, to shoulder the costs of either adapting to a warmer planet or perhaps making changes to the planet that counteract whatever climactic changes occur.

The plan for the rest of the paper is as follows. In “Some Stories of Ignorance” section, I introduce three different ways in which agents might become ignorant of climate change. In all three cases the agents fail to perform the same obligatory emissions-curbing action due to their ignorance. After discussing some shortcomings in present attempts to categorize ignorance in these cases as culpable or not, I move on in “Two Theories of Moral Responsibility for Ignorant Actions and Verdicts” section to examine two recent theories of culpable ignorance, and I show what these theories would entail regarding the culpability of the three ignorance types. “Upshots” section summarizes several theoretical and practical upshots. To foreshadow: the answer to the question posed in the title is “it depends…perhaps less often than you think”.

## Some Stories of Ignorance

I will argue below that the precise way in which ignorance about climate harms affects moral responsibility for causing such harms depends crucially on the details of the case. I think however that it is constructive to work with three general classes of ignorant agents because it is plausible that many actual cases of ignorance fall under this categorization scheme. Consider the following three cases.

- *Strategic ignorance* Strat is building a home. Like all other home-builders, he faces many, many decisions. He must choose the number of bathrooms, the color of the brick, the window size and so on. He is also faced with the choice of whether to implement an expensive pallet of energy-efficient technologies into the construction. Strat doesn’t follow developments about climate change carefully, but he knows enough to know that it might be something to look into. He knows for instance that he plans to live in the house for some time, and that the impact of implementing these energy-efficient technologies will be substantial. He also knows that if he asked his neighbor, who happens to be a well-respected climate scientist, whether there really are strong reasons to diminish one’s carbon footprint, she might give him evidence both that climate change is real and that he should opt for the green technologies. Because of the chance that his neighbor might tell him that things are quite dire and that he should definitely go green, he chooses not to ask and ignorantly goes with the cheaper and less efficient energy consuming construction.
- *Texas ignorance* Tex^7^ is also building a home, and he must make all the same decisions as Strat. One difference though is that he does follow developments about the causes of climate change avidly. However, the developments he follows are filtered through the slate of hyper-conservative news sources that he and generations of his family have always followed exclusively. Over the years this has lead to Tex’s strong disposition to be skeptical toward the findings of climate scientists, all of whom he takes to be brainwashed leftists who are hell-bent on propagating socialism through their environmental agenda. Despite all of this, Tex could come to know that climate change is real and that he should opt for the green technologies, but it would be very difficult. Ignorant that he should go green, he goes with the cheaper and less efficient energy consuming construction.^8^
- *Normal ignorance* Norm is also building a home, and he must make all the same decisions as Tex and Strat. Like Strat, he doesn’t follow climate science at all, but unlike Strat, it doesn’t even occur to him that he should look into it. Norm is either not that thoughtful about these kinds of decisions or he doesn’t believe that his actions will ever impact “bigger” problems. He could come to know that climate change is real and that he should opt for the green technologies, and it wouldn’t be at all difficult. Unlike Tex, Norm wouldn’t have to go against ingrained skepticism of anything he read about it. But, still Norm doesn’t look into it. Ignorant that climate change is a serious threat to human well being and, thus, that he should build green, he goes with the cheaper and less efficient energy consuming construction.

Strat, Tex, and Norm are all ignorant both about the threat posed by our changing climate and, derivatively, that they should take steps to diminish their carbon footprint, which in this case calls for utilizing energy efficient construction.^9^ They are ignorant about much else, not doubt, but it will do for the sake of what follows to focus on these bits of ignorance. The question of whether they are morally responsible for their ignorant actions can be addressed by inquiring into the related question of whether they are culpable for their ignorance. Although blameless ignorance seems to constitute an excuse, agents who are culpably ignorant seem to lack such an excuse. Such agents meet what’s called the epistemic condition of moral responsibility and can be blameworthy for their wrong actions despite their ignorance of some situation or state of affairs that is morally relevant and indeed their ignorance that they are doing anything impermissible.^10^

This issue has received relatively scant attention in the literature in climate ethics, and little of it has made contact with the developing literature on the epistemic condition of responsibility. Steven Vanderheiden has argued that these cases should be analyzed as negligence cases (2007). On such an analysis, ignorance would count as culpable if in coming to be ignorant, an agent falls short of some reasonable person standard. If someone fails to believe whatever a reasonable person would have believed then the ignorant agent believes negligently. Thus, if a reasonable person would believe both that climate change is occurring and that builders should take certain steps that mitigate contributions to it, then one might argue that agents like Strat, Tex, and Norm are negligent in their ignorance. Vanderheiden seems to embrace something like this line in the following:

Twelve years after the Rio Declaration committing developing nations to greenhouse gas abatement, and with three scrupulously researched and widely disseminated Intergovernmental Panel on Climate Change (IPCC) Assessment Reports, claims to reasonable ignorance concerning anthropogenic climate change are fully implausible, despite the uncertainties that remain in climate science. Even if the predictions about the harmful consequences of climate change turn out to be overstated, ignoring the considered recommendations of the vast majority of the world’s scientific community can only be described as willful ignorance, and cannot exonerate one from moral responsibility for resultant harm (2007, 91).

Vanderheiden’s appeal to a negligence analysis is instructive but underdeveloped. First, it does not suffice to establish the content of what a reasonable person would believe about climate science by pointing to the existence of a broad scientific consensus. This kind of consensus exists for many topics in science, yet we do not think it follows that non-scientists who are ignorant of consensus beliefs in these areas have beliefs that are not reasonable. Even in cases where much hangs on whether one’s beliefs accord with the scientific consensus (e.g. health risks of smoking and not exercising), the question of what the reasonable person would believe is at bottom a normative issue that is not settled by the mere existence of a consensus.^11^ Second, and relatedly, there is a lot of controversy about how the reasonable person standard should be set. According to some legal scholars, the reasonable person standard should be an objective one that abstracts from particular features of the agent in question and simply asks, given some situation and the relevant bodies of knowledge, what is it reasonable to expect anyone to believe or be aware of (Hart 1968)? Others think that no such objective reasonable person standard should set the boundaries of negligent action and belief formation. Instead, the standard should be subjectivized to certain features of the agent. This subjectivized standard would be influenced by the agent’s particular dispositions to believe and process evidence (Dressler 2001). According to an objective standard, Strat, Tex, and Norm might all be negligent in their ignorant failures to utilize energy efficient construction, but a subjective standard might result in a different verdict. Tex for example may be non-negligently ignorant given that he currently has the disposition to be skeptical about any claims made by scientists about climate change. It is plausible that ignoring them, rather than being an instance of willing and presumably objectionable ignorance, would constitute a reasonable, and indeed rational response to his evidence.^12^

A richer treatment of culpability for climate change ignorance is found in work by Daniel Fouk. He argues that the blameworthiness in ignorance cases can be established in two different ways. First, an ignorant agent will be blameworthy if her ignorance traces to a “benighting act”, which is a prior ignorance-producing action that the agent performs knowingly.^13^ If, for example, a physician knows both that a certain study has been published with important findings about the dangers of a popular drug and that for this reason she should read it but chooses not to read it, her failure to read it is a benighting act (or, in this case, omission).^14^ On this view if she were to harm a patient by prescribing this drug, then her ignorance about the dangers would not excuse her. A second way to establish blameworthiness in ignorance cases is to show that failing to become informed, the agent failed to engage in a certain “moral reflection”, about the permissibility of one’s actions.^15^ On this account, ignorance need not trace to a benighting action that was performed wittingly. Rather, one need only show that the ignorance was the product of a failure to reflect on what sort of information is relevant to the situation at hand. For example, if rather than thinking that she really should read that article, the physician just failed even to consider that it was important for her to do so, she would have failed to engage in the kind of reflection that can be expected given the stakes, which in this case are quite high. Her ignorance would be culpable and she would be blameworthy for her subsequent ignorant act of harming her patients, even though there was no unwittingly performed benighting action. Fouke argues that many agents who are ignorant about climate change would be culpably ignorant according to this second view. He notes that given the very high stakes, individuals who are ignorant about climate change ought to have engaged in the kind of serious inquiry and investigation that would have extinguished their ignorance. Strat and Norm, for example, simply ought to have undergone moral reflection regarding the impact of their decisions. The same would seem to be true for Tex, even though he is strongly disposed against conducting such a reflection.

Fouke’s discussion is helpful because, unlike Vanderheiden, he appeals to the literature on culpable ignorance and negligence. However, I think that more recent developments in this area cast doubt on the success of his treatments, especially as it concerns the latter way of establishing culpability. To anticipate the following section, the claim that agents have certain obligations to engage in moral reflection is yet another moral failure that can be performed ignorantly or not. If it is performed ignorantly, as it would be if the agent failed to realize that given the stakes she should reflect and investigate, then this is simply another ignorant failure to conform to one’s obligations. The problem has simply been pushed back a step. In the following section, I will carefully explicate two alternative ways of accounting for moral responsibility for ignorant actions, and I detail what these accounts would say about the cases of Strat, Tex, and Norm.

## Two Theories of Moral Responsibility for Ignorant Actions and Verdicts

The two most influential theories in the culpable ignorance literature are called “volitionist” and “quality of will” theories. Both theories emphasize the etiology of ignorance. According to the first family of views, blameworthiness for ignorant action must trace to blameworthy management of one’s beliefs, where the management of beliefs in taken to be a matter of performing certain investigative or reflective actions that are actual moral requirements. According to the second family of views, culpability for ignorance is determined by the ‘quality of will’ expressed by the agent who fails to inform herself. My goal of the section is to assess what the verdicts are of each theory for the three cases of ignorance discussed above.

### Volitionist Theories

Volitionism is an influential view recently developed by Michael Zimmerman (1997), Rosen (2004, 2008) and Neil Levy (2009). According to volitionism, agents are culpable for ignorant actions only if they trace to a particular type of witting belief mismanagement. According to Rosen, we all have certain “procedural epistemic obligations”, which, roughly, are moral obligations to engage in particular kinds of actions that have predictable epistemic upshots. Examples of such obligations include checking the expiration date on medications or checking one’s rearview mirror while backing out. Procedural epistemic obligations earn their normative force because compliance with them diminishes the likelihood of ignorance that, if acted upon, might lead to subsequent wrong or harmful actions. It is plausible that given the impact of certain of our actions on the climate that we have procedural epistemic obligations to investigate the magnitude of climate impact posed by our actions. Since the choice of construction will have is bound significantly to impact the carbon footprint of living in it, home-builders like Strat, Tex, and Norm have procedural epistemic obligations to investigate the impact of the various construction options they are presented with. If these agents have this procedural epistemic obligation, then the ignorance that traces to it is a candidate for culpable ignorance on the volitionist’s view.

Before moving on to a more careful analysis of these three cases, I must highlight another central feature of the volitionist’s position. This is the claim that culpable ignorance must trace to a *witting* failure to conform with one’s procedural epistemic obligations. The volitionist’s argument for this claim, which I alluded to above, is that it won’t do to say that an agent’s culpability rests only on her failure to conform to a procedural epistemic obligation. Assume that a given agent should have inquired into whether climate change was a real and worrisome phenomenon, but didn’t. We can ask if this omission was performed ignorantly or not. If it was not an ignorant omission, that is, if the agent believed that she should have looked into whether climate change was a real and worrisome phenomenon, but nevertheless chose not to do so, then she non-ignorantly fails to conform with her procedural epistemic obligations. On this basis, volitionists maintain that such an agent would be culpable for her ignorance about the reality of climate change.

If, on the other hand, her failure to look into whether climate change is real and worrisome was also performed in ignorance—if the agent didn’t take herself to have the obligation to look into this—then her ignorance-perpetuating failure to conform to her procedural epistemic obligations is itself an ignorant omission. Thus, we can only establish that an agent is culpable for *this* failure to look into things (and for the subsequent ignorance about climate change) by tracing the agent’s ignorance about her obligation to look into things to some still prior procedural epistemic obligation to investigate or reflect. The relevant prior procedural epistemic obligation might involve the kinds of inquiry that would be conducive to believing that one ought to look into the issue of climate change. Perhaps one ought to look into engaging in serious inquiry about the most serious ethical issues that we currently face as a global community. The result of such an inquiry, may well be the formation of a belief that one ought to investigate the reality and seriousness of global climate change, which is the procedural epistemic obligation of which we have been assuming our agent is unaware.

Imagine, now, that our ignorant agent also failed to conduct this ‘meta-inquiry’. Again, this failure itself will either be ignorant or not. If it is not—if the agent actually believed that she should engage in serious inquiry about what kinds of inquiry she should engage in—then she will be culpable for failing to know that she should inquire into the climate change issue and, thus, she will also be culpable for failing to believe that it poses a threat to well-being. If an agent knowingly fails to conduct some procedural epistemic obligation, even at this higher level, then she will be culpable for the resulting ignorance. If, on the other hand, her failure to conduct this meta-inquiry is also an ignorant failure, as it would be if she failed to believe that she ought to engage in serious inquiry about the most serious ethical issues that we currently face as a global community, then we have pushed the problem back yet another step. According to volitionism, her culpability for her ignorant failure to conduct this meta-inquiry (i.e. to investigate whether she should investigate whether climate change is real) must rest on some still prior procedural epistemic obligation, namely the obligation to investigate whether one should investigate whether one should investigate whether climate change is real. The only way to halt this regress and establish that someone is culpably ignorant for some belief and for subsequent ignorant omissions and their effects is to locate a knowing or witting failure to conform to one’s procedural epistemic obligations. Absent some knowing failure to perform one’s procedural epistemic obligation at some point in the etiology of the ignorance, the question will arise whether their failure to know that they had some procedural epistemic obligation was itself ignorant. Culpability for ignorance can only be established by ruling out this further question. As Zimmerman puts it:

“Every chain of culpability is such that at its origin lies an item of behavior for which the agent is directly culpable and which the agent believed at the time at which the behavior occurred to be morally wrong” (2010, 176)

Now that the outlines and motivation of volitionism are clear, it is time to see how it would classify the three cases of ignorance introduced above. Start first with Norm. Recall that Norm could come to know that climate change is real and that he should opt for the green technologies, but it simply does not cross his mind to look into things. If it failed even to cross his mind that he should examine the question of whether climate change is occurring, then he does not *knowingly or wittingly* fall short of any procedural epistemic obligation to investigate the reality of climate change. Note that it does not follow from the fact that it did not cross his mind to investigate the matter, that he lacked the procedural epistemic obligation to do so. We can assume that Norm has this obligation, and we can even assume that complying with it would have been relatively easy given the abundance of available information. Still Norm’s falling short of his procedural epistemic obligation does not suffice to make his resulting ignorance culpable. Volitionists can only ground culpable ignorance in witting failures. It follows that his ignorance is non-culpable, and thus that he is not blameworthy for his ignorant failure to utilize green construction materials nor presumably for all the ensuing and easily avoidable emissions. The same result would seem to hold for Tex. He is far from believing that he ought to examine the question of whether climate change is occurring, given that he already believes explicitly, on what may seem to others like very shaky grounds, that it is not occurring. At no point does he knowingly fail to investigate the climate change issue. And, given his history, it is not that he comes to his skepticism about climate change, which is fueled by a slate of regularly reinforced false beliefs, via any kind of knowing or witting belief mismanagement. We can assume that Tex, like many others who form their beliefs in what might be called poor epistemic neighborhoods, thinks he is thinking carefully, and that he is taking no witting epistemic risks. Thus, volitionists would hold that Tex is also non-culpably ignorant about climate change.

Before moving on to discuss Strat’s strategic ignorance, it is worthwhile to pause and take stock what volitionism entails generally for culpable ignorance about climate change. I chose these cases because they plausibly typify the way in which many people come to have their false beliefs about climate change. Norm is like anyone else affected by incuriosity or basic insensitivity to what is a highly significant problem facing all humans. Many individuals simply do not bother themselves with concerns beyond those near and dear. Effects that are not tangible may not register as compelling enough to warrant anything approaching careful consideration. People who are struck with this kind of incuriosity or insensitivity to their role in realizing harms brought about by climate change may suffer a deficit of intellectual virtue, and indeed they may even harbor intellectual vice. This does not suffice, however, to establish culpability on the volitionist’s view, unless these deficits were developed in the knowledge that they are risking ignorance about morally significant facts, that is in the knowledge that the agents are violating procedural epistemic obligations. For any case where this kind of witting belief mismanagement does not occur somewhere in the etiology of the false belief that climate change is not occurring, the agent in question would not be culpably ignorant.

Tex’s situation is distinct from Norm’s, but it is no less recognizable. Indeed, in certain regions, it may be just as common. Unfortunately, the public discussion about climate science in these regions is dominated by well-funded, badly informed and loquacious purveyors of falsehoods who are skilled at packaging their views in ways that make them seem legitimate, even to careful observers.^16^ Climate scientists and well-informed journalists are in the unfortunate situation of having to combat these typically politically motivated forces as they try to get the truth out. Even well-intentioned, truth-seeking individuals who find themselves in this milieu may find it difficult to navigate the competing voices. If this is the environment within which Tex formed his beliefs about climate change, then it should not be surprising that he comes out a skeptic who does not believe that he has any obligation to look any further into the matter. It is plausible of course that over time Tex comes to believe that he should reexamine his grounds for skepticism. If this were to occur, and if he opted not to do so, then he would be on the hook for on volitionist grounds. Absent this, however, it’s plausible then to think that Tex and people like him are not blameworthy for their ignorance. Moreover, on the assumption that being blameworthy for ignorance is a necessary condition for being blameworthy for ignorant action, he and his ilk would not be blameworthy for any failures to mitigate climate harms that are predicated on that ignorance.

Strat’s case of strategic ignorance is trickier to analyze in the volitionist’s framework. In the given case description, it is not clear whether Strat believes that he should investigate whether climate change is real. It could be that he indeed believes this, in which case he would be culpably ignorant. This culpability would be consistent with claiming that he is blameworthy for his ignorant failure to take steps toward climate change mitigation. However, Strat’s hesitation about asking his neighbor about climate science might stem from his own belief that it would be good or perhaps prudent to investigate whether antrhopogenic climate change is occurring. One may take oneself to have (really) strong reasons to look into things without taking oneself to have an obligation to do so. If this were the case for Strat, then the volitionist’s condition would not be met and so neither Strat’s ignorance and his ignorant action would be blameworthy.

It follows, given the volitionist’s necessary conditions on blameworthy ignorance, that few agents would be culpable for their ignorance about climate change. This result stems from the relative scarcity of witting belief-mismanagement in the context of climate change. It’s plausible to suppose that, like Norm, Tex, and one way of reading Strat, people normally construct their raft of false beliefs by conducting their epistemic affairs in ways they themselves fail so see as flawed. The implications this view has for the question of individual moral responsibility for climate change associated harms is significant. It shows that even if the issues of establishing a causal link between individual action and climate harms are solved, it may still be the case that many, if not most individuals would fail to be morally responsible for failing to mitigate climate change, given that they act from ignorance that is not culpable.

### Quality of Will Theories

One prominent way of responding to the volitionist is to argue that moral responsibility for ignorance and ignorant action can be established by showing that the ignorant agent displayed a lack of good will. On such views, an agent’s blameworthiness rests on the quality of her will and need not trace back to witting belief mismanagement. Quality of will theorists maintain that agents are responsible for ignorant actions only if their actions reflect their normative standpoint, where this standpoint expresses an insufficient lack of concern. Although this view has many proponents and variations the details do not matter for present purposes.^17^ Nomy Arpaly defends an influential version of this view according to which agents are blameworthy for their actions to the degree that their actions stem from a lack of good will (2002, 129–132). On her view, to say that an agent acts from a lack of good will is just to say that the agent fails to respond to the relevant moral reasons. Applying such views are relatively straightforward in non-ignorance cases. Suppose I know that climate change is a serious moral issue and that I know that I should utilize green materials in my construction projects. If I nevertheless choose not to utilize green materials because I find all the other options are aesthetically more desirable, then I fail to respond appropriately to relevant moral reasons—they are not sufficiently moving for me.

Quality of will theories can yield clear verdicts in cases of ignorant actions as well, though it can be trickier. For example, Arpaly discusses the case of Solomon, a sexist whose false beliefs about how women deserve to be treated are perpetuated by his ignorance about certain facts about women. She imagines that Solomon was raised in a milieu that stunted his moral development by sheltering him from certain facts that would make it more difficult for his sexist beliefs to persist (2002, 103–4). On her analysis, if this sexist escaped this environment and was confronted daily with evidence that women have all the morally relevant qualities that men have, we would expect him to change his tune. If his belief that say, ‘women are less intellectually capable than men’ were to survive even in the face of many obvious counterexamples, then Arpaly suggests that this manifests a lack of good will. This agent’s ignorance about these factual matters is often classified as ‘motivated ignorance’ (Moody-Adams 1994) and it is based on an agent’s failure to be sufficiently concerned to form accurate beliefs about the issue.^18^ Though the details of various quality of will views matter, it suffices for my purposes to adopt Arpaly’s version and see how it applies to the cases. The central question about the cases is whether Norm, Strat, and Tex are relevantly similar to Arpaly’s Solomon. Is their ignorance that climate change is a serious threat the manifestation of a failure to respond to relevant moral reasons? In other words, are they insufficiently concerned with the issue of climate change in a way that would make them culpable for their ignorance about climate change?

Before bringing the quality of will view to bear on the three cases, it is instructive to briefly contrast this approach with the volitionist’s. The starkest contrast is that the quality of will theorist need not trace ignorance to an instance where the agent knew she should investigate something but simply chose not to. For this reason, it does not produce the troubling implication that agents are only very rarely blameworthy for ignorant action. One other important point of contrast is that quality of will views can more straightforwardly account for the judgment that someone is blameworthy to some degree or other. The volitionist requires that an agent’s blameworthiness for ignorantly contributing to climate change must trace to the witting mismanagement of belief. If this necessary condition is met, then there is one less barrier to thinking that an agent is blameworthy for her ignorant action. It is not clear, however, how we could read off of this the claim that the agent is blameworthy to some degree. By contrast, one feature of quality of will views is that the degree of blameworthiness of ignorant agents is a function of the degree to which their ignorance manifests a lack of good will. Since the latter admits readily of degrees, the former should as well. The thought here is that an ignorant agent might manifest a greater deficit of good will, if he would be ignorant in counterfactual worlds where the evidence to which she fails to respond is stronger and more salient.^19^ As I will argue below, this feature of quality of will views may enable one to make elucidating distinctions between the cases.

Let’s start again with Norm. Does his failure to investigate climate change express or manifest a lack of good will? More precisely, does the fact that it never crosses Norm’s mind to look into the seriousness of climate change indicate a failure to respond to the moral reasons that underlie an obligation to do so? These reasons presumably have to do with *inter alia* the various threats to human well being posed by climate change. There are various explanations for why Norm might fail to respond to these reasons. One possible explanation for Norm’s failure to respond to reasons to investigate is that he has a standing belief that he lacks the time or ability to affect ‘big problems’, such as climate change. This might contribute to his disposition to find any reason to read about or otherwise investigate climate change less salient. Call this version of the case Ineffectual-Norm. We can imagine that, for similar reasons of feeling causally impotent, Ineffectual-Norm also does not vote or otherwise participate actively in the democratic process where he lives. On the basis of this belief, he’s adopted a general policy of just putting his head down and focusing on what seems to him to be tangible problems. When faced with the option of greener construction, he might dismiss it immediately on grounds of cost, his budget being one of the spheres within which he can manifest tangible effects. A similar tendency also results in his failure to believe that he should look into climate change. For him the thought of delving into the issue seems moot given that it’s a ‘big problem’ for ‘big, powerful people’ to sort out.

A second way of filling in the details is more straightforward. Imagine that Norm is just totally thoughtless about the effects that his actions might have. In this version of the case he doesn’t even have a standing belief that his actions are likely to be ineffectual. He simply fails to consider the downstream impact of his choices and focuses instead on the here and now. Call this Dense-Norm. Though both Norms fail even to consider whether climate change is really a threat, the verdict that the quality of will theorist should come to depends on which of these explanations holds.

Does Ineffectual-Norm express a bad quality of will? Norm’s ignorance about his obligation to investigate climate change is predicated on the belief that he can do nothing to combat climate change. The question for the quality of will theorist is then whether Norm’s forming this belief and his subsequent failure to investigate climate change express a lack of good will. There are readings of this case that are consistent with a negative answer. Ineffectual-Norm’s modesty with respect to what he can accomplish through his actions may be an honest mistake, rooted in nothing more than an understandably low estimation of his relative significance in affecting matters on such a large scale. Every time he gives into this tendency and fails to investigate, it may be regrettable, and we might even be justified in each case in intervening to try and change his mind. But, to attribute a lack of good will for this seems unjustified.

On another reading, however, Ineffectual-Norm’s modesty might seem to shade into an objectionable kind of complacency or stubbornness, either of which would indeed constitute a lack of good will. In Arplay’s discussion of Solomon, she notes that should his false beliefs about what women are capable of persist even after he has relocated to a society where he is exposed to many counterexamples, Solomon would reveal himself to have an objectionable lack of concern for getting things right with respect to morally relevant empirical beliefs about the capabilities of women (2002, 104). Solomon and perhaps this second version of Ineffectual-Norm would, through their ignorance, show that they have the quality of will that entails that they are culpably ignorant.

Dense-Norm raises similar issues but in a different way. It never even occurs to him to think about his abilities with respect to affecting climate. He is like the foodie who, despite ample reason to believe that omnivorism contributes to much avoidable animal suffering, simply does not ever take it seriously enough to look into it and may reside in a community in which the welfare of animals raised for food is simply not the topic of much discussion. Though it may be difficult for readers of this journal to believe, some individuals really may simply never stop to think about climate change. Dense-Norm may never consume media in which it is mentioned, and he may not be part of social networks in which it is brought up as a topic. In these respects, he would resemble Solomon quite closely. If this is right, then Dense-Norm’s ignorance may not trace to a lack of good will after all. Rather, he resides in a milieu in which even someone with admirable moral concern may fail to investigate and thus fail to know about climate change. On the quality of will view, this version of Dense-Norm would be blameless. However, also like Solomon, should Dense-Norm’s ignorance persist after repeated exposure to evidence that climate change is an issue of significant moral importance, then his incuriosity would begin to reflect a lack of good will.

It’s possible, though that Dense-Norm’s dispositions have more pernicious roots. Perhaps his incuriosity with respect to the science and moral significance of climate change is an aspect of a general self-centeredness that makes him wholly or mostly unresponsive to considerations that motivate even the most modest inquiry into both near- and long-term effects of his actions. Such a systematic lack of concern might in fact be an indication of a lack of good will after all, and would be consistent with the judgment that Dense-Norm is culpably ignorant after all. The difference between this version of Dense-Norm and the previous one is that one would expect his ignorance to persist even if he were to become aware of a possible link between his actions and climate change. His self-centeredness would wall off any avenues of inquiry, whereas his dense but not self-centered counterpart may well respond to what are now very salient reasons for inquiry.

This discussion of four versions of Dense-Norm reveals the care with which one must bring a quality of will approach to bear on specific cases of ignorance. The question of whether Dense-Norm’s failure even to consider issues about climate change and the potential contribution of his actions to it indicates a lack of good will, can only be determined by carefully understanding why the issue is not on his radar. Different explanations will yield different characterizations of his quality of will and, thus, different judgments as to Norm’s culpability for ignorance about climate change.

The application of quality of will theories to Tex is also somewhat tricky. Recall that Tex inhabits an epistemic bad neighborhood. Climate change skeptics are plentiful and effective at getting their message out. This context certainly plays some role in explaining Tex’s beliefs about the reliability of climate scientists and his attendant ignorance about climate change. The central question for the quality of will theorist, however, is whether holding these beliefs and being ignorant about climate change are manifestations of a lack of good will. As before, the details matter. It is certainly possible that another factor explaining Tex’s ignorance is a genuine lack of good will, say, toward people with fancy college degrees. Perhaps he simply fails to see warnings by scientists as anything more than missives from over-educated, self-important ‘readers’, and he has closed his mind to reconsidering this attitude. Though it’s true that it is easier for Tex to be Tex in his current environment than it would be were he to reside in a region that was less awash with propaganda, Tex’s ignorance in this case seems clearly to derive from a lack of good will. According to this interpretation, Tex would qualify as culpably ignorant. The sources are available to him, and he has the capacity to consume them (given that he readily consumes conservative media sources who are perpetuating misinformation). Note, however, that it would be difficult for Tex to see through all the white noise and come to hold true beliefs and, because of this, one might be inclined to think that his culpability is somewhat mitigated. Although the question of precisely how difficulty affects blameworthiness is too broad to take on in this paper, it is plausible to think that difficulty does mitigate blame in some contexts, and it is clear that Tex, and many of his compatriots must navigate a much tougher road to the truth than many others.^20^

The case of Strat receives the most straightforward application of the quality of will view. One of the most straightforward ways of displaying lack of concern for something is to act recklessly with respect to it. Since Strat is sufficiently informed about the possible threat of climate change, and since he knows his neighbor is a reliable source of further information about it, it is hard not to classify his failure to inquire as reckless risk-taking. The fact that he’s willing to take this risk is telling. He would rather ignorantly potentially contribute to an extremely serious problem than come to believe that climate change is real, a belief, the holding of which would make it more difficult for him to simply go for the cheaper, less-green construction materials. This displays a straightforward lack of concern for the moral issue at hand, and suffices to ground his culpability for ignorance on the quality of will view.

## Upshots

This extended discussion of Strat, Tex, and Norm has greater relevance than merely cataloging what volitionists and quality of will theorists are committed to saying about a collection of hypothetical cases. The three cases, together with their variants, cover a wide spectrum of ways in which agents might come to be ignorant about what many take to be one of the most important moral issues we currently face. For this reason, it is instructive to know when the two theories converge in their judgments and when they do not. The volitionist and quality of will views of moral responsibility both entail that agents who engage in strategic ignorance are culpable for their ignorance, and presumably, for their ignorant actions.

Where they diverge, however, we perhaps have reason to be less confident in the judgments they issue and in our own judgments about the divergent cases. The theories diverge for cases like Tex in which the ignorant agents reside in societies that for some reason or other are unfavorable having veridical beliefs about climate science and thus about one’s moral obligations with respect to mitigating it. On the one hand, and this is the volitionist’s point, they certainly don’t knowingly avoid reliable sources of information, given that their influences have painted them as unreliable. On the other hand, and this is the quality of will theorist’s point, such agents seem to display a lack of concern by failing to look sufficiently carefully at the data and for being overly dismissive of what are in reliable sources of information. This mixed verdict shows, at a minimum, that there is an important difference between strategic ignorance cases, where the two theories converged, and cases where reliable sources of information are obscured, which lead to divergent verdicts.

Convergence is only partial in cases in which ignorant agents are just unthoughtful about climate change. When their unthoughtfulness does not derive from complacency or stubbornness (as it did in the second variant of Ineffectual Norm) or self-centeredness (as it did in the the second variant of Dense Norm) then unthoughtfulness might be an understandable and honest mistake (as it seemed to be in the first variants of the Ineffectual and Dense Norm). These would be non-culpable ignorance cases on both accounts of culpable ignorance. However, when an agent’s unthoughtfulness is tied to these vices, then they would be culpably ignorant only according to a quality of will account; volitionists would still think their ignorance is non culpable.

It is beyond the scope of this paper to defend a particular theory of culpable ignorance and discuss what it entails about agents who are ignorant about climate change. My more modest goal was to enrich previous discussions of this issue with recent influential developments in work on the epistemic condition of moral responsibility. One important upshot from this discussion is that among the woefully large set of agents who are ignorant about climate change, there are different verdicts that one can come to as to culpability. Another is that there may be strong reasons to resist high degrees of confidence in our judgments given the number of relevant factors and their subtlety. A safe, but still important conclusion one may draw is that culpable ignorance about climate change may be widespread enough that we are often warranted in blaming individuals for ignorantly contributing to the realization of climate harms. But, we may just as often be misguided in directing our blame at certain non-culpably ignorant individuals. If we want to avoid making the same mistake that we are accusing such people of making, namely of acting from ignorance that is culpable, we should be sure that we get all the relevant information before proceeding to blame them.

Getting clarity on these issues also has broader implications for climate ethics for at least two reasons. First, the issue of whether individuals who are contributing to climate change are blameworthy is surely relevant to the project of putting the theories developed by climate ethicists into practice. The various innovative theories discussed in “The Problem” section give us the resources to sort individual actions into the categories of right or wrong. But, surely it is just as important to know of the actions categorized as wrong, which of them are performed by agents who are the fair targets of our reactive attitudes. If many agents are non-culpably ignorant about the reality and relevance of climate change, then though we have the theoretical justification to say that what they did was wrong, we are not in a position to hold it against them. Although Van de Poel et al. frame the following worry a bit strongly, at least as far as the excuse of ignorance is concerned, their point is an important one:

(I)t might very well be possible that no one can be fairly held blameworthy for certain collective harms. In as far as retribution for such harms is considered morally important, this gap might be considered morally problematic (2011, p. 65).

Second, one important way of responding to this possibility and avoiding or at least minimizing the problem that Van de Poel et al. call a “gap in responsibility distribution” (p. 65) is to reduce the instances of ignorance about climate change. Rather than amounting to a trite recommendation, a strong duty to reduce ignorance would accompany any other theory that posits the existence of individual obligations. Something that sets climate ethics apart from other fields of ethics is that it often requires actions that require significant sacrifice and thus go beyond what common-sense ethics might recommend. If these demands are stringent, then it would not be surprising if compliance was low.^21^

## Footnotes

1 cf: Vanderheiden (2007); Sinnott-Armstrong (2010).

2 Elizabeth Cripps has argued that Kagan’s argument does not apply in the case of climate harms because there is a disanalogy between the kinds of cases Kagan discusses and the climate change case (2013, 121–123). While I do not dispute that Kagan’s chicken market example (p. 121–128) does not closely map onto the way in which individual actions might make a difference in the realization of climate change-related harms, his air-pollution case (p. 129) is a closer fit.

3 For a defense of the claim that it is potentially problematic to view climate change as a collective action problem, see Hiller (2011) and Hourdequin (2010).

4 On the obligation to contribute to collective efforts to combat or mitigate climate change, see also van de Poel et al. (2011). For other defenses of the existence of individual moral obligations to address climate change see: Hourdequin (2010), Hiller (2011), and Morgan-Knapp and Goodman (2014).

5 It may be objected that the kind of empirical claims at issue differs from the kind of claims that are typically under discussion in the culpable ignorance literature. In her *locus classicus* “Culpable Ignorance” Holly Smith’s examples involve ignorance about everyday factual matters rather than claims, such as those at issue, which are at bottom hypotheses that are supported by scientific inquiry. This distinction is far from irrelevant given that the way that I avoid being ignorant about, say, whether my neighbor’s dog is behind my car as I’m backing out is very different from the way I avoid being ignorant about, say, whether vaccines are harmful. In the former case, I simply need to look and see, and in the latter I need to defer to reliable epistemic authorities, which may be more difficult to do responsibly. Avoiding ignorance about climate change would, for most people, involve identifying reliable epistemic authorities. The fact that this is harder to do than simply looking and seeing seems relevant to the question of whether someone who defers to unreliable sources is culpably ignorant. Although a complete treatment of this issue is beyond my scope, these issues do arise in “Quality of Will Theories” section below.

6 For a deeper and more general discussion about the relationships between these two senses of responsibility (and the various different senses of backward-looking responsibility) in the context of climate change, see (van de Poel et al. 2011).

7 Note that the author is a proud Texan and does not wish to impugn himself or any other Texan by using this example. The choice of Texas is motivated by the fact the congressional and senate delegation from Texas has more climate change deniers, both in number and in percentage than any other state. See: http://thinkprogress.org/climate-denier-caucus-114th-congress/#TX.

8 Fouke (2012, 120) discusses a similar example and argues that a Tex-like individual would be blameworthily ignorant because, despite the magnitude of the risks, he fails to be sufficiently diligent in educating himself. In the discussion below I discuss the problems with such a move.

9 Nothing of substance hangs on my choice of the particular obligation in this case. I chose it only because of the potential for very significant emissions reductions to be traced to this one decision. In this respect, it differs from the oft discussed and controversial case of taking a leisurely drive in one’s SUV. As I discuss in “Upshots” section, one might substitute the home-builder’s obligation to utilize low-emissions construction for any of the obligations that have been defended in the literature. For example, one could construct cases in which the agent’s ignorance about climate change leads them to opt against forming or joining some collective that will tackle climate change, which is what Cripps (2013) argues we as individuals are obligated to do. Also, though it may seem natural to read these as cases of having the forward-looking responsibility to use efficient materials, the question in this section is whether they are morally responsible in the backward-looking sense for failing to do so.

10 The literature on the epistemic condition of moral responsibility is flourishing. For an overview, see Robichaud and Wieland (forthcoming).

11 For a discussion of the reasonable person standard as it would apply in the context of meeting the epistemic condition of moral responsibility, see Sher (2009, 100–104).

12 For a discussion of the pitfalls of appeals to the reasonable person standard in cases of culpable ignorance in discussions outside the legal context, see (Sher 2009, 97–104).

13 Fouke is drawing on Holly Smith's "Culpable Ignorance"(1983).

14 Assume that circumstances are such that it would be easy for her to do so.

15 Here Fouke draws from Steven Sverdlik's "Pure Negligence"(1993).

16 Just how bad the public discourse is in a given region is an empirical matter, and so it is an open question just how representative Tex’s context is. To get some idea about how prevalent climate-change deniers are in the national and local media in the United States, the website Media Matters has compiled over 1685 instances of climate change science being called into question since 2004. See: http://mediamatters.org/issues/climate-change. Also relevant here is the fact that one of the two main political parties is comprised overwhelmingly of vocal climate change deniers, and it stands alone among conservative political parties as “an anomaly in denying climate change” (Båtstrand 2015).

17 Though there are many defenders of this kind of theory, a recent one is (McKenna 2012).

18 Here I am glossing over subtle issues about how we come to know when an agent displays an insufficient responsiveness to moral reasons. For a detailed treatment of this issue see King (2009, 584).

19 For a detailed attempts at fleshing out how quality of will accounts admit of degrees of blameworthiness, see Arpaly (2006) and Nelkin (2016, 361–366).

20 For recent explorations of the effect of difficulty on moral responsibility, and blameworthiness in particular, see (Bradford, forthcoming). The issue of whether blameworthiness comes in degrees is relevant topic that is increasingly discussed. See Coates and Swenson (2012) and Nelkin (2016). For my purposes, I assume that difficulty can impact the degree of one’s blameworthiness.

21 Of the different kinds of individual moral obligations discussed in “The Problem” section and fn 4, there are none for which non-compliance is not widespread.
